# Identify travel and health factors influencing well-being of the older adults–a case study in China

**DOI:** 10.3389/fpubh.2023.1213499

**Published:** 2023-07-19

**Authors:** Yi Zhu, Qian Zhu, Yongfeng Ma, Shuyan Chen, Hongcheng Meng, Muhammad Zubair

**Affiliations:** ^1^Jiangsu Key Laboratory of Urban ITS, School of Transportation, Southeast University, Nanjing, China; ^2^Jiangsu Province Collaborative Innovation Center of Modem Urban Traffic Technologies, Southeast University, Nanjing, China

**Keywords:** older adults, travel well-being, embedded feature selection, Gini index, transportation mobility, accessibility, relative importance

## Abstract

**Objective:**

With the increase in aging populations worldwide, the travel well-being of the elders has gained attention. The objective of this study is to examine the nonlinear relationships between the well-being of the older people in China and factors associated with travel and health.

**Method:**

Based on the data collected in China, combined embedded feature selection and decision tree built by Gini index were utilized to screen for influential factors and to determine the importance of the features selected. Tamhane’s T2 was used to study the differences in the important factors among older people with different levels of travel well-being.

**Results:**

This study found that the travel well-being of older adults depends mainly on accessibility to public places, such as schools and medical facilities, and the availability of bus services. Out of expectation, the most important influential factor of travel well-being of older people is the distance from home to high school. This is related to the traditional Chinese concept of education. In addition, it was found that the body mass index is more important than self-perceived health as an influence factor of travel well-being of the elders in China. Social skills are important factors too.

**Conclusion:**

This study investigated various health-related and travel-related factors and their impacts on the travel well-being of older adults Chinese with the overall goal to improve the quality of life of the elders in China. The findings may provide a theoretical basis for the implementation of various transportation management and urban planning and design -related policies to improve the travel well-being of older adults in China.

## 1. Introduction

### 1.1. Backgrounds

China has the largest number of older people globally and has entered a period in which its population is aging rapidly. Not surprisingly, the aging level and aging growth rate significantly exceeds world averages. By 2030, China’s population aged 60 and older is predicted to soar to 360 million, accounting for a quarter of China’s total population. An aging population brings about associated problems. For example, older people often have poor or declining physical health and are more likely to suffer from chronic diseases than younger people (1, 2). They also have more difficulty achieving a certain level of mobility than the general population. A key societal aspiration is to improve the travel well-being of older adults by analyzing the current status of their travel well-being and exploring the influential factors and mechanisms that underlie it.

Sociologists, economists, and psychologists have undertaken extensive efforts to determine travel well-being factors. Numerous studies have proven that health, education, employment, personality, and family relationships significantly affect the travel well-being of the elders. Yet, only in the last two decades have the effects of travel and transport systems on travel well-being begun to gain attention from researchers. These researchers have found that travel behavior, travel well-being, mobility, and transport service facilities can influence the travel well-being of the elders either directly or indirectly (3–8). Compared with other age groups, elder population has evident group characteristics regarding travel behavior and health status. Chronic disease is more prevalent among older adults who are usually not diagnosed and undertreated for their low awareness (9). Being moderately overweight causes less figure trouble to the older people, increasing their survival rate (10). Older people have special age-related medical needs, and their participation in social activities is often curtailed because of mobility restrictions. Facilities within walking distance may have a more significant impact on their travel well-being. Most older people are retired and have more free time than younger people. They are more likely to travel for optional activities, visit friends, to seek medical attention and go shopping. They relatively pay less attention to travel time and travel mode than younger people (11). Therefore, specialized research is necessary to understand travel-related factors that may influence the travel well-being of the elders.

Investigations into factors that affect travel well-being usually rely on statistical models, which include ordered logistic regression, bivariate probit models, ordinary least-squares regression, multiple linear regression, and structural equation models (7, 8, 12–17). The main shortcoming of linear regression and logistic regression models is that they can only find the linear relationship between independent variables and dependent variables and ignore the interaction between explanatory variables. In real life, travel well-being results from multiple factors that interact with each other. For example, transport systems can play an essential role in improving the health of the elders in terms of the older adults’ mobility and access to medical facilities as well as social opportunities and events away from home. Active transport modes such as walking and cycling and walkable communities have potential health benefits for older persons. Obviously, statistical models are unsuitable for evaluating travel-related and health-related factors combined effects.

To solve this problem, researchers can perform the explicit nonlinear transformation of input features which machine learning algorithms good at. Machine learning algorithms often have a strong learning capability, making them suitable for use in various research fields. In this study, we used a decision tree to study the influencing factors of travel well-being of older people because a decision tree can achieve nonlinear segmentation of features, handle the multicollinearity of the feature well.^21^Furthermore, a decision tree can produce visual classification rules and provides strong interpretability. Most importantly, a decision tree can output the importance of features, thus making comparisons between variables more intuitive.

Based on Chinese data from the China Family Panel Studies (CFPS) that were conducted by the Institute of Social Science Survey (18), this study built a decision tree to study the nonlinear relationship between the travel well-being of elders Chinese and factors that relate to the dimensions of both transportation and health. In particular, we divided the older adults into five groups, which correspond to the five levels of travel well-being identified in the CFPS. We also used Tamhane’s T2 to study the differences in essential factors among the five groups. The overall conclusions that can be drawn from this study provide a theoretical basis for implementing of various travel well-being-related policies.

The organization of the remainder of this paper is as follows. In Section 2, we review the literature for definitions of travel well-being and its influential factors and presents the motivation for this research and the contributions of this paper. Section 3 discuss the data and methodology used in this study. Section 4 presents the model results. In Section 5, we analyze the critical, influential factors and suggest policies for improving the travel well-being of the older people in China. The final section draws vital conclusions.

### 1.2. Literature review

Scholars have undertaken a great deal of work to define travel well-being and explore the factors that influence travel well-being. We reviewed the literature related to definitions and determinants of travel well-being.

#### 1.2.1. Definitions of travel well-being

The term ‘travel well-being’ has taken on various subtle and different meanings over time. Philosophers first attempted to provide a clear conception of travel well-being 2000 years ago, which formed two theoretical camps: hedonic, purported by Epicurus, and eudaemonic, purported by Aristotle (19–21). The former regarded the most incredible pleasure as the source of travel well-being, while the latter proposed that ‘travel well-being is good’, which considers virtue and pleasure. Bradburn and Noll put forward the concept of ‘life satisfaction’, defined as constructing an estimator’s standard and comparing the estimator’s current life condition to an expected life condition (22). Andrew and Withey later used life satisfaction to measure travel well-being (23), which initiated their research into the cognitive model of travel well-being. Based on the cognitive model of travel well-being and hedonic travel well-being, other researchers proposed the concept of subjective well-being, which consists of three components: people’s emotional responses, domain satisfactions, and global judgments of life satisfaction (24, 25). Researchers who study eudaemonic travel well-being believe that subjective well-being is just one part of travel well-being and that too much emphasis is placed on emotions. They argue that self-expression is another essential component of travel well-being whereby an individual devotes themselves to study or work and give full play to his/her potential to obtain the experience and pleasure of self-realization (21, 22, 26). These two perspectives, hedonic and eudaemonic, constantly integrate with and complement each other (27, 28). Travel well-being generally reflects people’s eternal pursuit of a good life. In this work, because of the single-item question measuring travel well-being in the CFPS dataset, we refer to travel well-being as an overall evaluation of life-based on “intuitive, affective appraisal and cognitively guided evaluation” (20).

#### 1.2.2. Research on travel well-being of older adults

In recent years, researchers have begun to pay more attention to the effects of transportation and travel on travel well-being, and research objectives gradually have been expanded to include older adult populations (4, 5, 7, 29, 30). However, the precise relationship between the travel well-being of older people and travel remains unclear in China because of the relative lack of research. Early studies universally focused on the impact of health-related factors on the travel well-being of the elders, with little attention paid to the impact of travel-related factors. Furthermore, the travel environment, customs, culture, and health status of the older adults in China are different from those in Western countries. Directly apply conclusions of studies conducted in Western countries to a Chinese context is problematic. In China, walking and public transport are the preferred travel modes for the elders (11). Car ownership does not seem important to them. In addition, in Chinese culture that embraces family travel well-being, the older people often take responsibility for taking care of grandchildren (31). This unique family division of labor may change influencing factors of the travel well-being of elders in China.

#### 1.2.3. Determinants of travel well-being

##### 1.2.3.1. Effects of demographic characteristics

With such specification of conceptualizations of travel well-being, empirical researchers began to investigate the determinants of travel well-being. Travel well-being is affected by personal characteristics and other factors that change with age, including material conditions, social and family relationships, and social roles. By categorizing 71 articles that focus on the socioeconomic status of older people, Read et al. found that elders of poor socioeconomic status are concerned about their poor health and well-being (31). Later research also noted that education level indirectly effects travel well-being (32, 33). Concerning to the older adults in China, illiterate adults report significantly lower levels of travel well-being than literate adults. In addition, the level of education further affects subjective well-being indirectly via levels of income and health (16). Blanchflower showed that married women who have received higher education have the highest levels of travel well-being (34).Social activities and participation (e.g., providing support to others and participating in volunteer activities) and visiting friends also have been found to boost travel well-being and improve the health of older people (13, 35).

##### 1.2.3.2. Effects of travel-related factors

The current elders population (age 65+) is in better physical condition than previous generations, participate in more social activities, and has a greater need for travel (36).The movement of people to and within areas of activity where people can satisfy their recreational needs and enjoy events and public services is realized in transportation. Convenient transport systems can generate freedom and belonging to the broader community. Greater mobility can also increase older people’s confidence to achieve specific goals and improve their well-being (8, 37). Researchers have found that accessibility to transport service facilities (railway stations, bus stops, being close to the city center or schools) has a positive impact on travel well-being (7, 8). People who live close to the city center tend to have positive emotions, due partly to convenient transport that can carry them to shops or allow them to enjoy delivery services (38). Moreover, convenient transportation and a walkable community can improve people’s attitudes toward health and travel (37, 39, 40). However, according to research and analysis of older adults in the United Kingdom, walking difficulties can have a negative effect on travel well-being of the elders (3).

Researchers have analyzed a variety of outdoor activities, the choice of modes of transport, and the impact of achievable mobility on the subjective well-being of older people. The results indicate that mobility and physical activity have the most significant impacts on subjective well-being (41, 42). In conjunction with the importance of mobility, maintaining contact with friends can improve the subjective well-being of older people (35). Therefore, the effects of travel-related factors are intertwined with social factors and health-related factors.

##### 1.2.3.3. Effects of health-related factors

An inverted U-shaped curve between life satisfaction and age is evident during older adulthood. People’s life satisfaction increases with age and peaks at around age 65 (43). Some studies have pointed out that it is not aging that alters the level of travel well-being, but rather other factors, such as declining health, related to age groups (2, 7, 44). For example, in aging, older adults often experience limited physical ability and deteriorating health that may contribute to widespread depression and lower spiritual well-being (44, 45). A survey of elders in the United States showed that poor physical health has a significant and direct impact on the subjective well-being of older people (7). For example, chronic diseases reduce the quality of life and thus negatively affect travel well-being (3). Furthermore, people who have a high body mass index (BMI) or chronic lung disease are more likely to be depressed than those who do not (46, 47).Furthermore, compared with their objectively determining health status, the self-evaluation of the health of older adults is closely related to their spiritual well-being (44). Research also reveals the inverse relationship whereby travel well-being influences the health of the elders, with higher levels of travel well-being leading to a reduction in depressive symptoms. Some researchers have suggested that subjective well-being should be incorporated into health evaluation systems and considered in the development of medical systems (48). In short, researchers have found links between travel well-being and health, which includes self-perceived health, mental health, and physical health, in older adults.

### 1.3. Motivation and contributions

In the above, we review the literature for definitions of travel well-being and its influential factors. Personal circumstances, travel-related, and health-related factors have been found to be directly or indirectly correlated with the travel well-being of older adults. While previous studies have examined the factors that influence travel well-being from either a health or transportation perspective, few have examined the relative importance of travel and health factors together on the overall travel well-being of older adults. Moreover, most studies have relied on statistical models to investigate these relationships, with little application of machine learning algorithms to explore nonlinear relationships. Additionally, research has primarily focused on Western countries where the primary mode of travel is by car, making it crucial to examine the specific factors influencing travel well-being among older adults in China, given the country’s complex transportation environment and differences in travel behaviors.

This study aims to address this gap by investigating the travel-related and health-related factors that influence the travel well-being of older adults in China. Given traditional cultural values, older adults in China may be more likely to engage in family-related activities rather than personal leisure activities, and their travel modes may differ from those in Western countries. To achieve this goal, we utilize non-Western data collected in China and employ machine learning algorithms to identify and assess the significance of variables. Furthermore, through the combination of kernel density distribution and Tamhane’s T2, we provide targeted recommendations to enhance the travel well-being of different groups of older adults in China.

In summary, this study offers a unique contribution by investigating a rarely studied combination of travel-related and health-related factors that impact the travel well-being of older adults in China, using non-Western data and machine learning algorithms. It also takes into account the country’s specific cultural and transportation context, providing targeted recommendations for improving the travel well-being of different groups of older adults.

## 2. Materials and methods

### 2.1. Dataset

The data used in this study were derived from the CFPS aimed at investigating the government benefits that Chinese residents receive and many other topics, including transportation, educational outcomes, family relationships and dynamics, and health. The CFPS sample covers 25 provinces, municipalities, and autonomous regions. The interviews were launched officially in 2010 and were conducted in rural and urban areas simultaneously. Due to the complexity of the Chinese social system, the CFPS launched multidimensional data collection efforts at three levels: community, household, and individual. The community-level data include the political environment, village/residence, infrastructure, population, resources, transportation, revenues, and expenditures. The household-level data include family structure and membership, living conditions, social interaction, income and expenditure, and asset status. The individual-level data include education level, income, marital status, and psychological and physical health status. The profound research value and quality of the CFPS have been well recognized and acknowledged by the academic community.

This article selected data from the CFPS survey in third-tier cities (no subway) and rural areas to ensure that the overall categories of travel modes are relatively similar. And this study focused on the CFPS survey year 2010 because the data for 2010 contain more information about travel well-being, transportation, and health than the other survey years. The survey conducted in 2010 was initiated in March and lasted 4 months. Although the survey period from March to June covers two seasons, the mild and comfortable weather during spring and early summer, along with a stable temperature range, did not result in any significant deviations that may have affected the survey results. Therefore, the season did not have any impact on the survey results. No surveys were conducted in the northwest quadrant because most of these areas are plateaus, with vast areas and sparse populations, making it too difficult to conduct surveys.

We screened the data for the elder’s population aged 60 and above from the CFPS dataset and removed data records with missing values from the analysis. Furthermore, we removed records with unreasonable values (e.g., waiting for a bus for more than 70 min). The final sample was 3,820 participants between 60 and 109 years old (*M* = 68.16; SD = 6.863) of whom 54.74% were male and 45.26% were female.

The purpose of this study is to validate the effectiveness of data analysis methods. Considering the significant resource investment required for conducting comprehensive social surveys, university researchers are unable to perform such studies. Therefore, this study chose to use the authoritative CFPS dataset. This approach can be extended to current research on the travel well-being of older adults. The analysis method of this dataset has significant practical and theoretical significance for the development of care measures for the current older population (the older adult) in terms of their travel well-being.

### 2.2. Variables

The dependent variable in this study is travel well-being. Participants in the CFPS responded to the question ‘How would you estimate your level of travel well-being?’ on a scale of 1 to 5, with 1 indicating not happy at all and 5 indicating very happy. The reliability and validity of measuring travel well-being in single items are proven (48). The independent variables are travel-related factors and health-related factors.

Socio-demographic variables include gender, marital status, education level, income, personal assessment of social status [social status], and sociability; the latter corresponds to the question ‘How well do you think you get along with other people?’ These variables are the most common factors that influence travel well-being. We added two variables, area and region, because CFPS covered 25 regions and investigated elders in urban and rural areas. The geographic contexts and built environment of urban and rural areas of each city are pretty different. We specifically considered sociability to include social relationships and support that can mitigate loneliness and increase the travel well-being of older adults (49).

The travel-related factors can be divided into three categories: family travel information, public transport services, and accessibility to public places (schools, medical facilities, libraries, leisure facilities, shopping centers, etc.). Family travel information includes the most commonly used travel mode in daily life [travel mode] and private car ownership [ownership of car]. We combined bus and subway within ‘public transport’ unlike prior research studies that typically included five commute modes (walking, biking, driving, taking a bus, or taking a subway). Most of the cities in China did not have a subway in 2010; only 11 older adults (0.2%) in the sample had traveled by subway. We added two additional travel modes: electric bike and ‘other’. Worth noting also is that, in rural China, somewhat unusual means of transport, such as donkey carts and tractors, are commonplace. Public transport services are reflected in terms of the distance to the nearest bus stop [distance to bus stop] and average waiting time at the bus stop [waiting time], referring to the time between passengers arriving at the bus stop and getting on the bus, which represent the coverage and convenience of public transport, respectively. Public places accessibility is reflected in the time it takes to reach the nearest medical facility by the fastest transportation mode [time to the medical facility], distance from home to the nearest high school [distance to high school], and time to the nearest city/town commercial center using the respondent’s daily travel mode [time to commercial center]. These variables are treated as continuous variables except travel mode and car ownership. Note that the distance to the nearest high school is a new and rarely considered indicator of travel well-being of the elders. We selected this factor because high school admissions in China require proximity to a high school and the older adults are often the grandchild’s primary caregivers.

Like the travel-related factors, the health-related factors also can be divided into three categories: objective physical health, objective mental health, and self-perceived health. Objective physical health includes BMI and several physical health impairment indicators reflected in the following questions: ‘Have you been ill in the past 2 weeks?’ [sickness], ‘Have you had any chronic diseases in the last 6 months?’ [chronic diseases], and ‘Have you been hospitalized in the past year’? Note that BMI is an important international standard to measure degrees of weight and health and is computed by dividing the height by the square of the body weight. BMI is divided into five ranges by Asian standards, representing underweight, normal weight, overweight, and obese classes one and two. Mental health is measured by the frequency of the feeling that life is meaningless in the last month [meaningless], which is a question taken from the Center for Epidemiological Studies-Depression Scale (CES-D) (50). Self-perceived health is the subjective assessment of one’s physical condition [self-perceived health].

**Figure 1:**
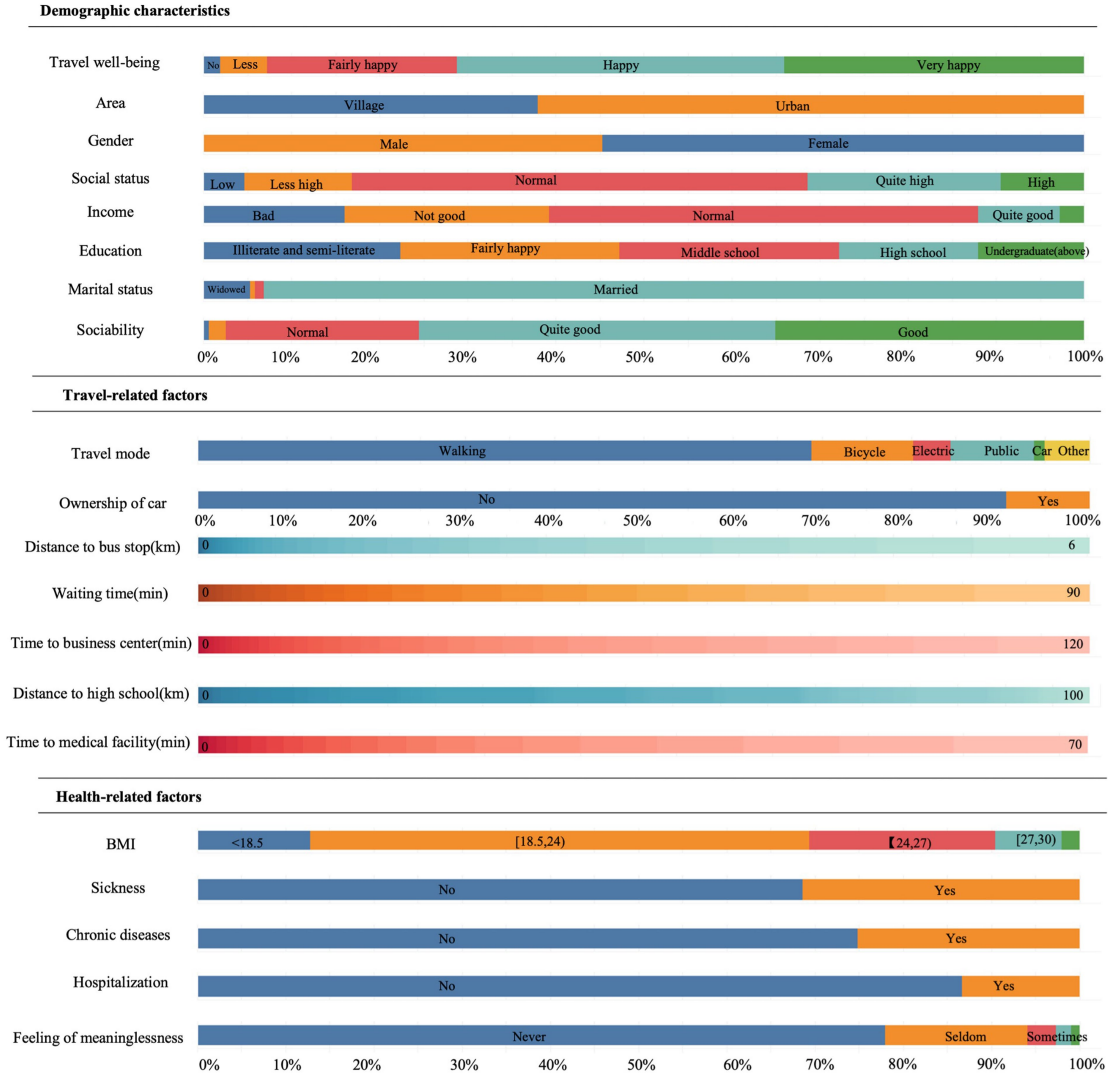
Share of discrete instances and range of continuous instances.

**Figure 2:**
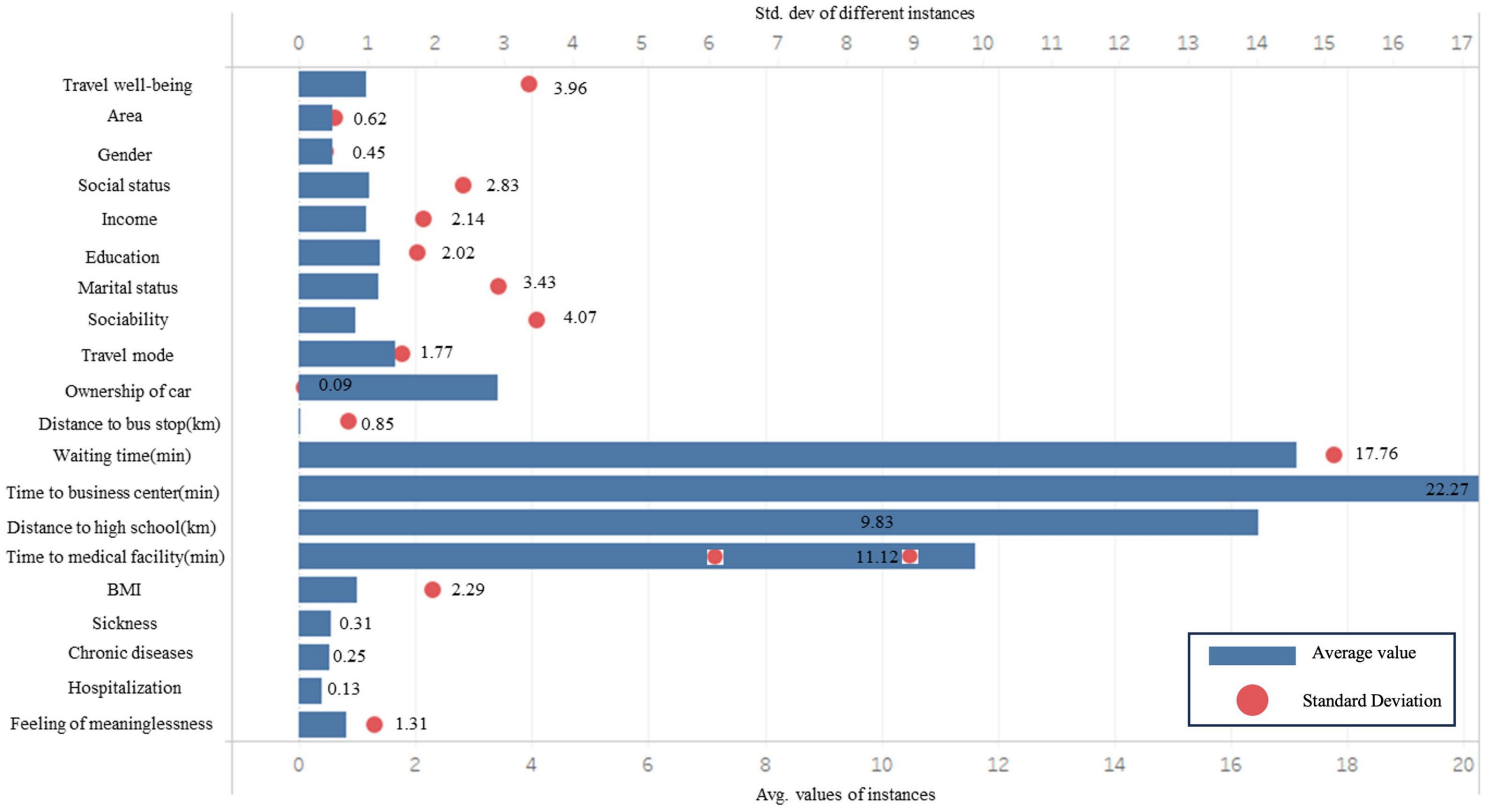
Mean and standard deviation of attributes.

Table 1 and Figures 1, 2 provides summary statistics for the travel well-being groups and the explanatory variables. The travel well-being values of the older people range from 1 to 5, with 1 indicating least happy and 5 indicating most happy. These five values correlate with this study’s five ‘travel well-being groups. Table 1 shows the three categories of the independent variables: basic demographic information about the individual, travel-related factors, and health-related factors. The table shows that the average Travel well-being value of the older adults is 3.95, which is close to 4, indicating that most elders tend to give relatively high scores for their travel well-being. The table also shows that only 9% of the older adults’ own private cars. The average distance to a bus station is 848 meters, which is more than 500 meters; China has been trying to improve the coverage of bus stops so that they are located within a radius of 500 meters. The average waiting time for a bus is long, 17.76 min, which is more time than most people are willing to wait. Only 13% of seniors were in the hospital during the previous year. A quarter of the elder respondents had chronic diseases and the proportion (31%) of older people who had fallen ill in the past 2 weeks is relatively high. Most older adults never think that life is meaningless and feel in good health. The data also show high scores for sociability.

**Table 1:** Variable descriptions.

Variable	Description	Mean	SD
Travel well-being (groups)	1 = not at all happy, 2 = less happy, 3 = fairly happy, 4 = happy, 5 = very happy	3.96	0.971
Demographic characteristics			
Region	0–24 represents the codes of 25 survey regions	11.53	7.687
Area	0 = village, 1 = urban,	0.62	0.485
Gender	0 = male, 1 = female	0.45	0.498
Social status	1 = low, 2 = less high, 3 = normal, 4 = quite high, 5 = high	2.83	1.019
Sociability	1 = bad, 2 = not good, 3 = normal, 4 = quite good, 5 = good	4.07	0.834
Income	1 = bad, 2 = not good, 3 = normal, 4 = quite good, 5 = good	2.14	0.988
Education	1 = illiterate and semi-literate, 2 = primary school, 3 = middle school, 4 = high school, 5 = undergraduate and above	2.02	1.174
Marital status	1 = widowed, 2 = divorced, 3 = unmarried, 4 = married	3.43	1.163
Travel-related factors			
Travel mode	1 = walking, 2 = bicycle, 3 = electric bicycle, 4 = public transport, 5 = car, 6 = other	1.77	1.399
Ownership of car	0 = no, 1 = yes	0.09	0.291
Distance to bus stop	Continuous variable (m)	848.88	1119.089
Waiting time	Continuous variable (min)	17.76	14.589
Time to business center	Continuous variable (min)	22.27	18.154
Distance to high school	Continuous variable (km)	9.83	14.033
Time to medical facility	Continuous variable (min)	11.12	9.894
Health-related factors			
BMI	1 = [0, 18.5], 2 = [18.5, 24], 3 = [24, 27], 4 = [27, 30], 5 = [30, 50]	2.29	0.858
Sickness	0 = no, 1 = yes	0.31	0.464
Chronic diseases	0 = no, 1 = yes	0.25	0.434
Hospitalization	0 = no, 1 = yes	0.13	0.339
Feeling of meaninglessness	1 = never, 2 = seldom, 3 = sometimes, 4 = often, 5 = almost every day	1.31	0.702
Self-perceived health	1 = poor, 2 = less healthy, 3 = fair, 4 = good, 5 = excellent	3.88	1.092

### 2.3. Screening factors that may influence travel well-being

We used embedded feature extraction, decision tree, and Tamhane’s T2 to explore some of the variables that influence travel well-being. First, to select the appropriate indices, we used an embedded feature extraction method based on Extremely Randomized Trees, a machine learning algorithm, to remove interfering factors and clarify the relationships between each factor and travel well-being. Then, we used a decision tree with the Gini index to obtain the degree of importance of the selected features and to determine each factor’s degree of influence on travel well-being. Finally, to analyze the role of the crucial factors at the group level, we combined Tamhane’s T2 with violin plots to investigate the differences in the critical factors among the five travel well-being groups.

Connections between things often can be seen more clearly by stripping away redundant factors and leaving only the key ones, which also can reduce difficulties associated with the analysis and computational burden of a model. Thus, features should be selected before further study. To this end, we used Select from Model, a feature extraction module built into Scikit-learn, an efficient machine learning tool, to extract features that contribute significantly to travel well-being. Select from model is a meta-transformer that can be used with any estimator with feature coefficients or feature importance attributes after fitting. If the corresponding feature coefficients or feature importance values are below the threshold provided, these features are considered unimportant and removed. Such removal is an embedded method for feature selection. The intrinsic estimator we chose for this purpose is Extremely Randomized Trees, which can calculate the importance of features during learning (51).

Distance calculations are used frequently in machine learning applications and statistics. However, different dimensions may lead to unreasonable results in distance calculations that depend on features with large dimensions. Standardization can eliminate the effect of the dimensions of the feature on the results without changing the feature’s original distribution. In this study, the standard deviations of the discrete variables’ (see Table 1) generally were between 0 and 1. In contrast, the standard deviations of the continuous variables were significantly higher, with the most minor being 2.408 and the most prominent being 2323.64. Therefore, we used Z-score standardization before feature extraction to scale the original variables’ data, thus making them conform to standard normal distributions (μ = 0, σ = 1). The steps are as follows:

Step 1: Use Z-score standardization to scale the original continuous variables data.

Step 2: Use the Select from Model module to screen the variables and select Extremely Randomized Trees as the intrinsic estimator.

### 2.4. Acquiring the importance of features

Decision trees are standard decision support tools that have been used for factor analysis and are used in machine learning to make decisions based on a tree-like structure (52). Compared with other machine learning models, decision tree results are explainable and the importance of features can be calculated. Quinlan’s work can explain the principles that underlie decision trees (53, 54). In a tree structure, each internal node represents a test of a feature, each branch represents a test output, and each leaf node represents a category. The key to decision trees is to choose the best partition attributes. The three main ways to measure the pros and cons of attribute classification are information entropy, gain ratio, and Gini coefficient values. The importance calculation is based on these coefficients. The difference in the calculation results between the Gini index and information entropy is negligible although the Gini coefficient can be calculated much faster than information entropy. Thus, we used the Gini coefficient calculate the importance of the features in this study. Gini(D) represents the probability that two randomly selected samples belong to different categories from set D. Gini index (D, a) represents the probability after segmentation based on feature *a*. The lower the Gini index value, the greater the purity of the sample. Knowing the value of the feature with the highest Gini coefficient value, the uncertainty of the travel well-being score decreases. In other words, the feature that leads to the most significant reduction of the Gini index value of the sample is correlated closely with travel well-being and is the critical important feature. The sum of the importance of all the features is 1. The steps are as follows:

Step 1: Calculate the Gini index value for each feature, as shown in Equations 1 and 2.

(1) $$\;Gini\left(D\right)=1-\overset{\left|y\right|}{\underset{k=1}{\sum }}p_{k}^{2}j$$

(2) $$\;Gini\_index\left(D,a\right)=\overset{V}{\underset{v=1}{\sum }}\frac{\left|D^{v}\right|}{\left|D\right|}Gini\left(D^{v}\right)$$

where $p_{k}^{2}$ is the proportion of the samples in category *K* in the current set D ( k = 1,2,3,…, |y| ); $D^{v}$ is all the samples of D contained in the branch of the v node whose value for feature a is $a^{v}$ ; and $a^{v}$ is the possible value of the feature a .

Step 2: Calculate the feature’s importance. The importance of the feature is computed as the (normalized) total reduction of the Gini index value that is yielded by that feature.

### 2.5. Obtain intergroup differences of important factors

We used Tamhane’s T2 to determine the intergroup differences in each variable and used violin plots to show the specific intergroup differences. Tamhane’s T2 is a non-parametric multiple comparison tool that gives a test statistic using the *t*-distribution (55).Tamhane’s T2 can be performed when the equal variance assumption is violated (56). The commonly used method for this purpose is the analysis of variance (ANOVA) that requires data to satisfy the homogeneity of variance. We found that our data failed to satisfy this requirement, so we used Tamhane’s T2 instead.

A violin plot is used to show the distribution and probability density of multiple data groups. It combines the characteristics of a box plot and density, plot and is used mainly to show the distribution shape of the data. The width of the violin represents the density, and three dotted lines define the locations of the quartiles. A violin plot is similar to a box plot but is better for showing density; hence, we used violin plots in this study.

## 3. Results

**Figure 3:**
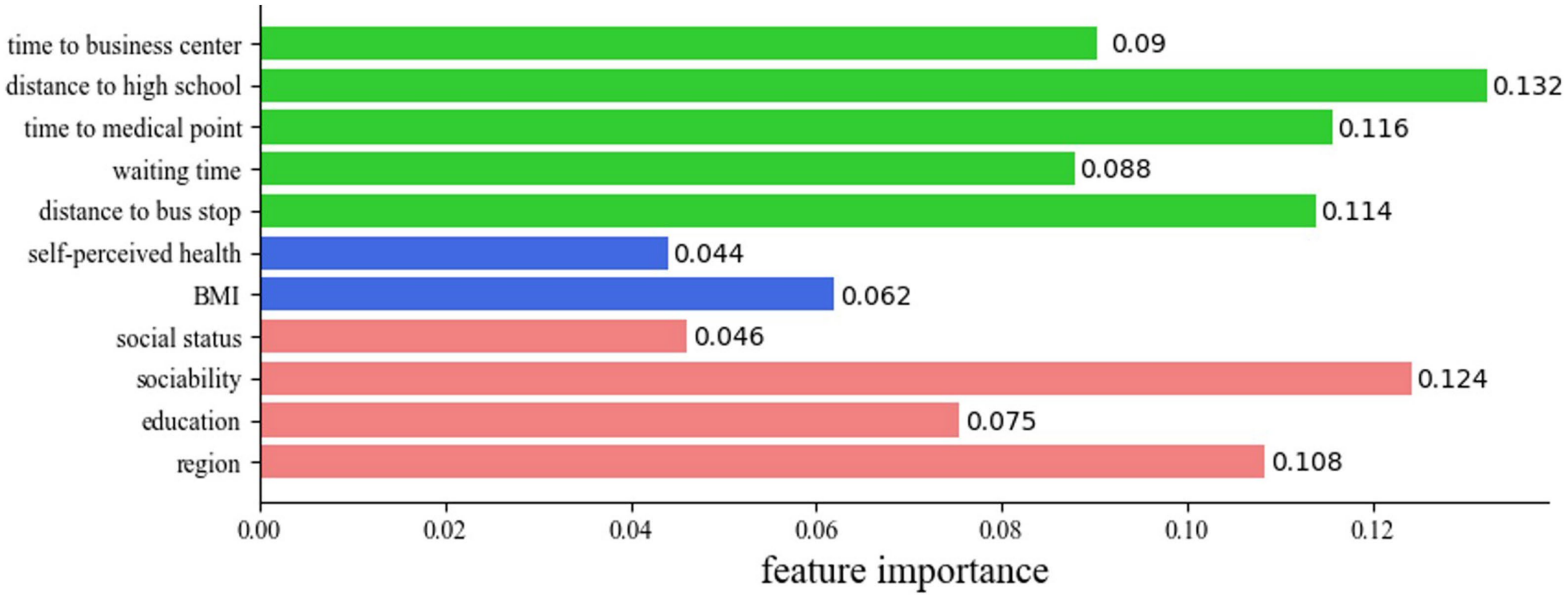
Importance values of explanatory variables.

We obtained the variables that can best predict travel well-being through feature selection and determined their importance using the decision tree. Figure 3 shows the 11 variables that contribute the most to predictions of travel well-being and thus were selected. The colors of the bars represent the categories of the variables. Green, blue, and red represents traffic-related factors, health-related factors, and demographic characteristics, respectively. The value indicates the importance of the variable to predict travel well-being. The higher the value, the greater the effect of the variable on travel well-being.

**Figure 4:**
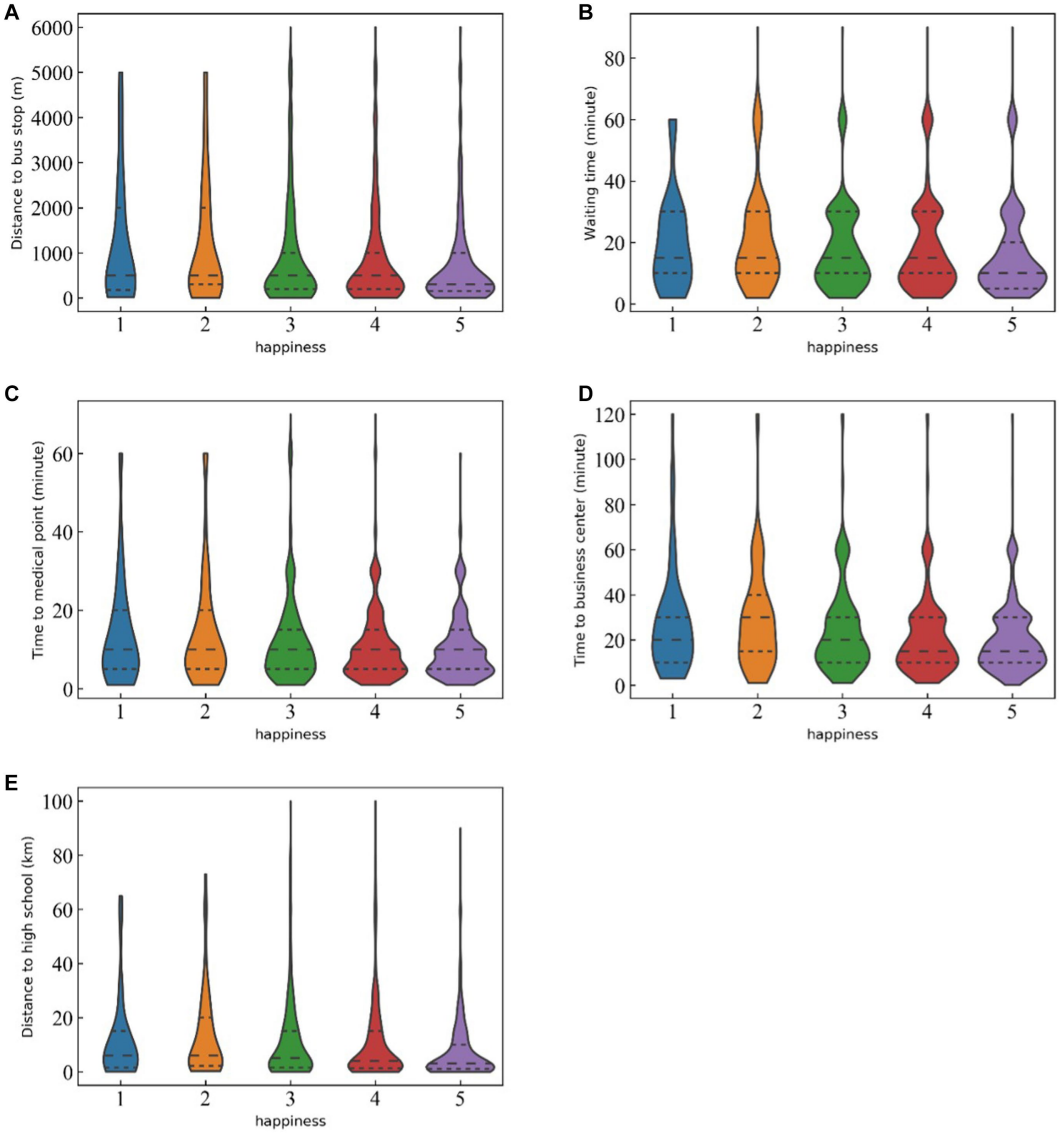
Density distributions of travel-related variables among groups.

Figure 4 shows violin plots of travel-related and health-related factors density distributions. The width of violin represents the probability density of distribution.

Some socio-demographic variables, such as social status, sociability, region, and education level, contribute to the Travel well-being of older people. The most immense contribution comes from sociability, which is defined loosely as the ability to gather with and get along with other people. Also, the province where the elders live is an important factor that affects their travel well-being. It has been observed that the elders residing in more developed provinces tend to report higher levels of travel well-being compared to those living in less developed provinces. We also found that income is not an important determinant of Travel well-being. Educational background and social status also are linked to the travel well-being of older people, which is in line with other research findings (33). We found no gender differences in the travel well-being of the older adults.

As shown in Figure 4, when looking at the effects of public places accessibility, the most important feature is the distance from home to high school, which is also the most important factor of all the travel-related factors. Figure 3 (e) shows that the distance to high school value distribution for Group 5 (highest level of travel well-being) is significantly different from that of the other four travel well-being groups (*p* < 0.01). A high proportion of elders are ‘very happy’ to live within 5 km of a high school. Also, the time to a medical center matters more to the older adults than to the nearest commercial center. About to time to a medical facility, the distributions of Group 5 and Group 4 do not differ significantly, but the kernel density distributions of these two groups are significantly different from those of Group 2 and Group 3. Time to a commercial center has the same pattern across groups. The happy elders also have good access to health care, shopping centers, and leisure facilities. Concerning public transport services, the older people are concerned more about the distribution of bus stops than the waiting time at bus stops. The distance to a bus stop is nearly as important to them as the time to a medical facility. The distance to a bus stop for Group 5 is significantly shorter than for the other travel well-being groups and is concentrated within 600 meters. Also, compared to the waiting time for other groups, the waiting time for Group 5 is significantly shorter, with 50% of the people waiting less than 10 min and 75% of the people waiting less than 20 min for a bus. Therefore, older people who can conveniently get to a bus stop or station that provides high departure frequency are more likely to report higher levels of travel well-being.

**Figure 5:**
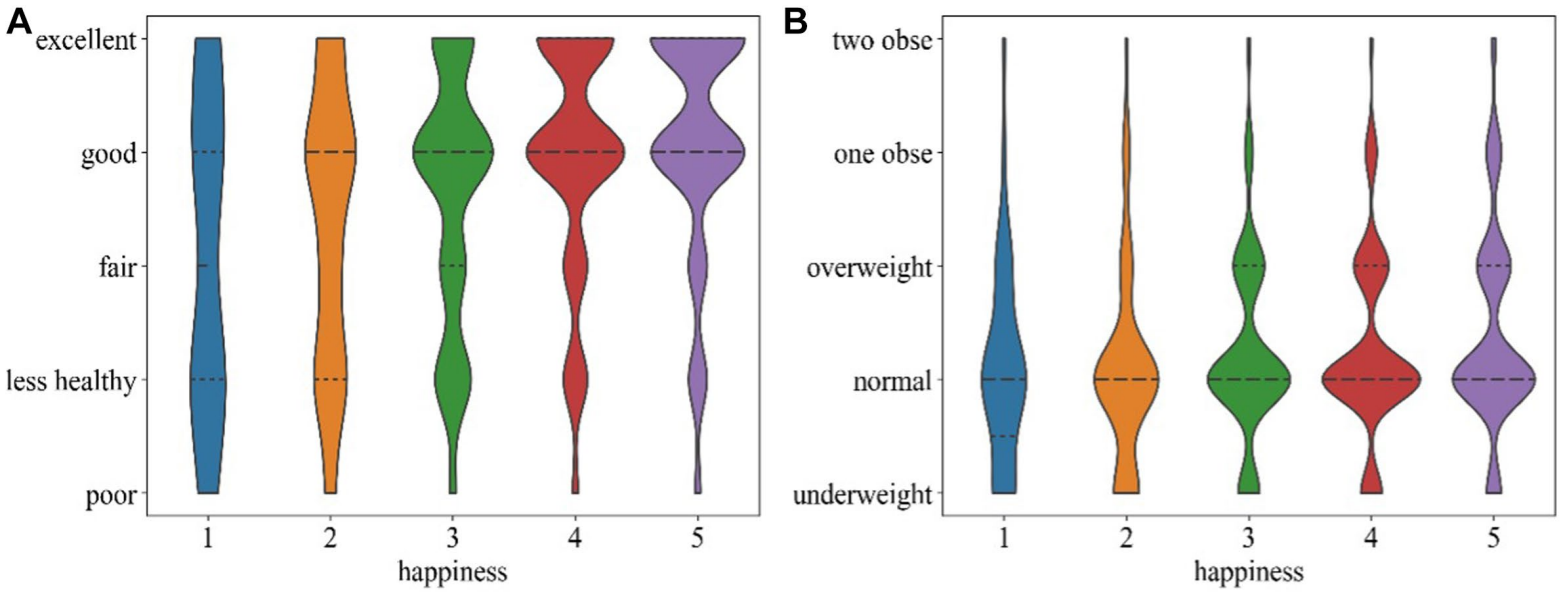
Density distributions of health-related variables between travel well-being groups: **(a)** self-perceived health and **(b)** BMI.

Similarly, Figure 5 shows the density distributions of the health-related factors. In terms of health-related factors, the results presented in Figure 5 (a) and (b) respectively indicate that self-perceived health, and BMI has important impacts on the travel well-being of older adults, and that BMI is more important than self-perceived health. As expected, and shown in Figure 5 (a), elders who perceive themselves to be in good health are more likely to report greater travel well-being. Figure 5 (b) shows that Group 5 in the normal BMI range is smaller than that of Group 4, but the proportion of older adults in the obese range is higher. Mental health factors were meaningless in this study, and other objective health indicators, including sickness, chronic illness, and hospitalization, had no important effects on travel well-being.

## 4. Discussion

In this study, we examined the contributions of different variables, especially transportation mobility and accessibility-related factors, to the travel well-being of an elders Chinese population. The results help to identify travel-related and health-related factors that have the greatest potential for improving the travel well-being of older adults. Older people tend to report high levels of travel well-being, with a mean value of 3.95 (18). The reasons for this outcome may be that they have lower expectations of quality of life and are less able to change low conditions, so they are more likely to be forced to adapt to the environment to maintain travel well-being compared to younger people (16, 27).

Older people’s capacity to get along well with others strongly effects their travel well-being. This finding also corresponds to the results of Pinquart and Sorensen’s study which show that quality social interactions can lead to higher levels of Travel well-being (35). The elders who have high emotional intelligence can participate in high-quality social activities to build their social network and gain support from the network. Other findings include that, except for being in a state of poverty, income has little effect on travel well-being (57). Also, as expected, elders with higher education and social status reported higher levels of travel well-being than people without such resources (58).

Transport and travel affect the travel well-being of older adults in many ways because transportation provides them with mobility and accessibility. This unexpected finding that the distance to a high school can be a travel well-being indicator seems to reflect that proximity to a high school benefits grandchildren of the elders, thus enhancing the travel well-being of grandparents. In China, living near a high school is one of the requirements for most high school admission. Children and grandchildren of older people living near schools are more likely to have access to an enjoyable learning atmosphere, good educational resources and make great achievements. The development of younger generations may have a more important impact on the elders travel well-being than hitherto thought. Moreover, the elders living near the high school can take care of the school-going grandchildren more conveniently, which relieves the burden on their children and let them enjoy the family travel well-being (37).

The time that it takes to travel to a medical center also is related to the travel well-being of the older people, and we found this factor to be more important than the time it takes to travel to a commercial/shopping center. This finding is not surprising, because the older adults decreasing physical functioning means that proximity to medical facilities is more important to them than proximity to shopping and entertainment areas (2). Also, mobility in emergencies, especially when outbreaks of disease occur, provides psychological benefits to older people (59). Being able to reach healthcare facilities quickly allows older people to obtain timely medical treatment. In addition, although not as important as the ability to reach a medical center quickly, the time to the nearest city/town commercial center also affects the travel well-being of the elders where commercial centers, shopping and entertainment are concentrated. Corroborating this observation, previous studies have indicated the importance of mixed land use and retail and services in the neighborhood of older adult people’s dwellings (60). This underscores the substantial influence of easy access to a variety of facilities on the travel well-being of the older adult population.

The distance to a bus stop and the waiting time at a bus stop also affect the travel well-being of older people. The importance of the distance to a bus stop might be explained from two perspectives. First, compared to Western countries where most families have a private car, less than 10% of older adults own a car in China. When seniors need to travel a long distance, taking the bus is their first choice. Moreover, the government provides public transport subsidies for older adults so that the older people can take the bus or transfer to another bus for free. Therefore, having a bus stop near their residence is significant to the elders. By contrast, the farther away they live from a bus stop, the lower their travel well-being level associated with taking the bus (8). In a previous study conducted in Japan, it was found that living closer to public transportation was associated with higher levels of physical activity among older adults (61). Therefore, bus stops or stations that can be reached quickly and provide high departure frequency offer convenience for older adults and enhance their overall travel experience.

Unexpectedly, we found that the mode of travel does not have an important impact on older people’s Travel well-being, which differs from the findings of other studies. In our findings, walking is the first choice for most elders (67.67%) for daily travel, followed by cycling (11.22%). The reason for this finding is that older people’s family members are concerned about their safety and do not want them to ride bicycles or electric vehicles which are prone to accidents and require a high level of attention. Furthermore, considering the low levels of vehicle ownership in third-tier cities and rural areas, the travel options of older adults are limited. As results, unlike younger people, older people are forced to choose these two travel modes. Also, given the compromised physical condition of many older adults, they might not be able to enjoy walking or cycling. They might even feel that those modes of travel limit their ability to travel long distances. Zhu and Fan found that walking is associated with four negative emotions (62). Morris and Guerra’s study indicates that utilitarian walking has no relationship with total well-being (63). Another reason is that the unbalanced structure of travel mode data reduces this factor’s ability to predict travel well-being. This understanding aligns with the discussions in two World Health Organization documents - ‘Global age-friendly cities: a guide’ and ‘Measuring the Age-Friendliness of Cities: a guide to using core indicators’ (60, 64). These documents underscore the importance of proximity to public transport and favorable walking conditions. They also advocate for a walking distance of less than 500 meters to public transport. Such recommendations dovetail with the principles of Transit-Oriented Development, which similarly emphasize the importance of short distances to public transport. Therefore, these documents and the present findings jointly illustrate the crucial role of easily accessible public transport in the well-being of older adult travelers.”

Our finding that car ownership is not an important factor is consistent with Deka’s research that likewise found that automobile ownership does not effect on travel well-being (25). In 2018, the motorization level in China stood at 173 motor vehicles per 1,000 inhabitants. In contrast, the European Union has a significantly higher average, with 567 passenger cars and 83 commercial vehicles and busses per 1,000 inhabitants. Therefore，the motorization level of China is very low compared to that of Western countries. According to the CFPS, only 9.18% of elders own a car and 1.13% of the older people’s preferred travel mode is by car (including private cars and taxis). In China, only 1.98% of people over 60 have a driving license (11). Drivers over the age of 70 are required to undergo a yearly physical examination and are not allowed to drive if their physical condition is not up to standard. In addition, young people generally think it is dangerous for older people to drive and prevent them from driving even if the older adult has a valid driver’s license. Therefore, car ownership has a very limited effect on improving the travel well-being of the elders in terms of their mobility.

Concerning health-related factors, this study found that BMI and self-perceived health have an important effect on the Travel well-being of older people; these results are supported by previous studies (10, 64–66). Linna et al. (67) found that a parabola curve exists between BMI and subjective well-being, and that subjective well-being is optimal in the overweight category. Being slightly overweight can reduce the mortality of older adults. However, obesity can lead to various diseases and decrease travel well-being (10). Self-perceived health is strongly associated with travel well-being among older adults. This finding confirms the results of previous studies that report a significant relationship between self-perceived health and psychological well-being among older adult (44).

Based on our analysis results, we propose several suggestions to improve the travel well-being of older adults in China. First, sociability is the most important personal factor that impacts travel well-being, which indicates that targeted transportation policies may result in the increased travel well-being of people who currently are severely restricted from maintaining social support networks because of problems associated with the transport system. Previous Studies found that some common barriers that older people face when traveling, such as lack of knowledge, physical limitations, lack of social support and negative beliefs of fear (68, 69). Therefore, a barrier-free travel environment is essential for older people to get out of the house and socializes. Second, improving the service level of public transport should be a government priority. Bus networks, stations, and stops should be planned and located so that the elders can walk to a convenient bus stop or station. Third, policy makers should pay more attention to unhappy people because the needs of those who are unhappy are more urgent than for those who are happy. The data show older adults who are unhappy take a longer time to the nearest medical facility than those who are fairly happy. Therefore, we propose combining transportation planning and land use, synchronizing medical resources and medical staff, setting up medical facilities in every township and community, and ensuring convenient transportation channels to medical facilities (70). Lastly, a reasonable school admissions policy (based on comprehensive test score rather than the school district of residence) may improve the travel well-being of the older people.

## 5. Conclusion

Conducting empirical research and examining how travel and health influence the travel well-being of the elders are necessary and important across various contexts. For social equity, governmental and institutional policies should be tilted toward those who need help most urgently. To ensure that policies serve to improve the travel well-being of older adults Chinese with low levels of travel well-being, the gap between them and the older people who feel happy needs to be understood. Given the rapid increase in China’s aging population, the factors influencing the travel well-being of elders Chinese are important considerations and worthy of study. This study investigated various health-related and travel-related factors and their impacts on the travel well-being of older adults Chinese with the overall goal to improve the quality of life of the elders in China.

This study aimed to investigate the contributions of travel and health-related factors to the travel well-being of older adults in China. We found that sociability and social connections are the most important personal attributes affecting travel well-being, while income does not have a significant impact.

Regarding accessibility to public places and other travel-related factors, the distance to the nearest high school was found to be the most important determinant of travel well-being for older people. This discovery is a new and interesting one that merits additional qualitative research to understand why proximity to school may have a significant impact on the travel well-being of older adults Chinese. We also found that the time to reach a medical facility is more important to elders Chinese than the time to a commercial center. Both the distance to a bus stop and the waiting time at a bus stop are important factors for travel well-being, and that the distance is more important than the waiting time. From the health-related perspective, BMI levels and self-perceptions of health have an important effect on the travel well-being of older adults.

Surprisingly, travel mode did not significantly influence travel well-being. The reason for this finding is that older people’s family members are concerned about their safety and do not want them to ride bicycles or electric vehicles which are prone to accidents and require a high level of attention. Furthermore, considering the low levels of vehicle ownership in third-tier cities and rural areas, the travel options of older adults are limited.

This study has limitations. Limited by the CFPS dataset, the travel well-being dimension in this paper is relatively singular. In addition, the CFPS sample data cover 25 provinces/cities/autonomous regions that have substantial differences among them. This paper does not consider the influences of locational factors on the travel well-being of the elders. Additional research that entails a detailed survey of the travel well-being of the older people in specific regions may be useful. It can help people better understand the specificities of the populations of certain provinces, and can indicate the importance of methodological application in other sociocultural contexts, of developing countries.

Few studies have examined the combined effects of travel and health on people’s travel well-being, and the relationships remain ambiguous. Using a large sample of Chinese older people, we were able to explore the influential factors of travel well-being among health and travel. Ways to maintain older adults travel well-being by improving their health and travel should be analyzed comprehensively. While this study has limitations, the findings provide evidence for associations among various factors and put forward policy changes and new questions for further research. As China’s aging population continues to grow, it is crucial to understand the factors influencing the travel well-being of older adults and develop policies that aim to improve their quality of life. To improve the travel well-being of older adults in China, policymakers should focus on developing a barrier-free travel environment that allows elders to socialize and access public transport conveniently. Additionally, bus networks, stations, and stops should be planned and located in a way that facilitates access for older adults. It is also essential to improve the synchronization of medical resources and medical staff to provide timely medical treatment to the elders, especially those who are unhappy.

## Data availability statement

The original contributions presented in the study are included in the article/supplementary material, further inquiries can be directed to the corresponding author.

## Author contributions

SC and YM: funding acquisition, supervision, writing-reviewing and editing, and critical revision. YZ: conceptualization, methodology, data curation, writing-original draft preparation, and writing-reviewing and editing. QZ, HM, and MZ: conceptualization and methodology. All authors contributed to the article and approved the submitted version.

## Funding

This research was funded by the Humanities and Social Science Foundation of the Ministry of Education of China with Grant No. 20YJCZH121 (Recipient: YM).

## Conflict of interest

The authors declare that the research was conducted in the absence of any commercial or financial relationships that could be construed as a potential conflict of interest.

## Publisher’s note

All claims expressed in this article are solely those of the authors and do not necessarily represent those of their affiliated organizations, or those of the publisher, the editors and the reviewers. Any product that may be evaluated in this article, or claim that may be made by its manufacturer, is not guaranteed or endorsed by the publisher.
